# Left Ventricle Architecture and Valvular Integrity Following Microaxial Mechanical Support: A Two-Year Follow-Up Study

**DOI:** 10.3390/jcm10061273

**Published:** 2021-03-18

**Authors:** Georgios Chatzis, Styliani Syntila, Harald Schuett, Christian Waechter, Holger Ahrens, Birgit Markus, Dimitar Divchev, Marc Rogmann, Konstantinos Karatolios, Georgios Bouras, Bernhard Schieffer, Ulrich Luesebrink

**Affiliations:** 1 Department of Cardiology, Angiology and Intensive Care, Philipps University Marburg, 35043 Marburg, Germany; syntila@med.uni-marburg.de (S.S.); haraldschuett81@yahoo.com (H.S.); christian.waechter@staff.uni-marburg.de (C.W.); holgerahrens@yahoo.com (H.A.); birgit.markus79@gmail.com (B.M.); dimitardivchev@yahoo.com (D.D.); marcrogmann@yahoo.com (M.R.); konstantinoskaratolios@yahoo.com (K.K.); bernschieffer@yahoo.com (B.S.); ulrichluesebrink81@yahoo.com (U.L.); 2 Yale School of Medicine, Yale University, New Heaven, CT 06510, USA; bourasgio@gmail.com

**Keywords:** cardiogenic shock, protected-PCI, Impella, valvular integrity, aortic valve, mitral valve

## Abstract

Although the use of microaxilar mechanical circulatory support systems may improve the outcome of patients with cardiogenic shock (CS), little is known about its effect on the long-term structural integrity of left ventricular (LV) valves as well as on the development of LV-architecture. Therefore, we aimed to study the integrity of the LV valves and architecture and function after Impella support. Thus, 84 consecutive patients were monitored over two years having received Impella^TM^ CP (*n* = 24) or 2.5 (*n* = 60) for refractory CS (*n* = 62) or for high-risk percutaneous coronary interventions (*n* = 22) followed by optimal medical treatment. Beside a significant increase in LV ejection fraction after two years (*p* ≤ 0.03 vs. pre-implantation), we observed a statistically significant decrease in LV dilation (*p* < 0.001) and severity of mitral valve regurgitation (*p* = 0.007) in the two-year follow-up period, suggesting an improved LV architecture. Neither the duration of support, nor the size of the Impella device or the indication for its use revealed any devastating impact on aortic or mitral valve integrity. These findings indicate that Impella device is a safe means of support of LV-function without detrimental long-term effects on the structural integrity of LV valves regardless of the size of the device or the indication of support.

## 1. Introduction

Cardiogenic shock (CS) is a clinical condition of systemic hypotension secondary to cardiac dysfunction with adequate or elevated filling pressures, leading to inadequate perfusion and subsequent failure of end-organs [1]. Mechanical circulatory support (MCS) devices, such as the transvalvular microaxial pump Impella (Abiomed Inc., Danvers, MA, USA), are emerging as a treatment strategy leading to a reduction in afterload and thus improving cardiac reserve and ventricular metabolism to maintain hemodynamic stability, augmentation of cardiac output (CO), reduction of catecholamine doses and subsequently improvement of perfusion of vital organs [2,3,4,5]. By continuously drawing blood from the left ventricle (LV), Impella unloads the LV decreasing its work and myocardial oxygen demand and results to an increase in mean arterial pressure (MAP) and CO, leading simultaneously to a decrease of catecholamines and its’ devastating effects to microvasculature [6]. The net result of all these effects is an improved systemic perfusion and increased coronary flow decreasing at the same time pulmonary wedge pressure and right ventricular afterload. At the same time, the evolution of percutaneous coronary intervention (PCI) has witnessed unprecedented advances in the past two decades allowing interventional cardiologists to attempt revascularization of more complex coronary anatomy in patients often declined for surgical intervention, since multiple comorbidities, advanced age, or poor distal targets make surgery often an unattractive option. Such patients, mainly with multi-vessel or left main coronary artery disease and severely depressed LV function, may be considered for high-risk PCI. However, these patients present to have no reserves and transient ischemia caused by coronary balloon or stent inflation may result in hemodynamic collapse or lethal arrhythmias. Impella disposes an effective tool to protect the patient from hemodynamic decline and to allow for recovery of hibernating or stunned myocardium [7,8,9] and has been demonstrated to apply the necessary to support in this group of patients, while it presented to have a better long-term outcome compared to IABP in the setting of protected-PCI [7,10,11,12]. Due to the fact that Impella needs to cross the aortic valve (AV) and that it is being positioned on the mitral valve (MV) inflow tract, concern has been raised about the changes caused on the valves of the left heart as well as to proximal aortic or left ventricular (LV) bed. To the best of our knowledge, up to date there is only one study focusing on the valvular integrity of the left heart valves after Impella support [13]. However, this study was performed only in a protected-PCI setting, included only patients supported with Impella 2.5 and studied the outcomes in a follow-up period of only three months. Moreover, data concerning the long-term effects of Impella unloading on the LV architecture and on the left heart valves are lacking. Therefore, we aimed to evaluate for the first time the long-term safety and effects of support with Impella 2.5 and CP on the architecture of the left heart.

## 2. Materials and Methods

### 2.1. Study Design

We retrospectively analyzed data from all patients supported with Impella 2.5 or CP recruited to our institution (Department of Cardiology, University of Marburg, Marburg, Germany) from September 2015 to June 2020. Indications for the MCS was protected-PCI setting or refractory CS. Protected-PCI was recommended for hemodynamically stable patients with a combination of severe coronary artery disease (the vast majority left main or proximal left ascending artery lesions), and other conditions such as diabetes, peripheral vascular disease, history of angina, or prior surgeries and with an ejection fraction of <40%. All cases were presented in our multidisciplinary heart conference and decision was made upon all available data with colleagues of the cardiothoracic and anesthesia department together with the patient. Patients with refractory CS receiving another support device in the same period with Impella were excluded from the analysis. Refractory CS was defined as the need for continuous infusion of vasopressors to maintain systolic blood pressure >90 mmHg with evidence of end-organ hypoperfusion such as cool extremities, altered mental status, and/or serum lactate >2.0 mmol/L. Coronary angiography and PCI were performed in a conventional manner. Patients were treated with drug-eluting stents and/or percutaneous transluminal coronary angioplasty. The extent of coronary revascularization and adjunctive therapies were left at the operator’s discretion.

The study was approved by the local ethics committee of the Philipps University of Marburg. The need for informed consent was waived due to the retrospective nature of the study. The study adheres to the STROBE guidelines for observational studies.

### 2.2. Patients’ Management

All Impella devices were implanted through the femoral artery and placed via the retrograde approach through the AV into the left ventricle under fluoroscopic control in catheterization laboratory. The implantation procedure, the post-implantation therapy and extraction of the device were based on standard protocols and standard operating procedures developed through initial experience with the Impella device from February 2013 till August 2015. All out of hospital cardiac arrest patients were treated with targeted temperature management (mild hypothermia of 33–34 °C for 24 h followed by gradual rewarming to 37 °C in hourly increment of 0.25 °C) with an endovascular cooling device (Thermogard Temperature Management System, Zoll, Chelmsford, MA, USA), intending to maintain temperature below or equal to 37 °C until 72 h after cardiac arrest. In all patients, inotropes and vasopressors were used to obtain a mean arterial pressure ≥ 65 mmHg. In patients with Impella support, flow was adjusted to maintain mean arterial pressure ≥ 65 mmHg with the lowest possible dose of catecholamines and to cover metabolic needs as assessed by central venous oxygen saturation (≥70%) and serum lactate levels (<2.0 mmol/L). All patients were treated with a standard medical protocol concerning infections. The decision to wean the circulatory support device was based on resolution of shock and clinical assessment. Weaning process was performed by gradually decreasing Impella support. Once the support of the device was reduced to low levels (performance level 1) with stable mean arterial pressure ≥65 mmHg, no or low doses of catecholamines, central venous oxygen saturation ≥70% and serum lactate levels < 2.0 mmol/L, the device was removed in intensive care unit (ICU) and hemostasis was achieved with mechanical compression (St. Jude Medical FemoStop) according to standard protocol. In patients with protected-PCI the Impella was extracted either direct after end of coronary intervention in the catheterization laboratory or in the ICU. In the latter setting, the decision to extract the device was made, when a hemodynamic stability without malignant arrhythmias together with decreasing cardiac markers of LV overload were observed.

### 2.3. Echocardiographic Data

Echocardiograms were obtained in a standardized protocol before, direct after Impella implantation, on the day before scheduled discharge and on follow-up in the outpatient clinic. In patients with protected-PCI and planned direct device removal, an additional echocardiogram was obtained in the catheterization laboratory before and after extraction. Only patients with follow-up echocardiographic data of at least 4 months were included in the analysis, since the main aim of the study was the long-term valvular integrity after Impella support. Assessments focused on structural integrity of heart valves and myocardium and LV systolic function. Demographic information was removed from each echocardiogram for blinding purposes. The echocardiograms were analyzed by 3 Level III cardiologists, which underwent and passed stringent testing for acceptable inter- and intra-observer variability prior to commencing analysis of study echocardiograms. The variability thresholds were after testing the cardiologists in 150 random echocardiographic tests showing a smaller than 5% variability on the interpretation of the main echocardiographic data presented in this survey. Furthermore, each cardiologist underwent an intra-observer test with demonstration of 100 random echocardiographic findings in 5 separate controls. A variability of <5% was set as acceptable. Valve structure and function were evaluated by standard methods and according to current guidelines established criteria [14,15]. Mitral regurgitation (MR), mitral stenosis (MS) as well as AV regurgitation (AR) and stenosis (AS) severity were assessed from multiple views qualitatively with quantitation relying on an integrated assessment of all available information. For the better visualization of the valve integrity or valve failure as well as for the determination of the ejection fraction (EF) additional 3D images were acquired, an additional transesophageal echocardiography was carried out by every moderate or severe valve stenosis or insufficiency in the initial hospital stay as well as in the follow-up period. All echocardiographic analyses were performed with the Philips EPIQ 7 ultrasound device.

### 2.4. Data Collection and Study End-Points

Intra-hospital clinical data, outcomes and follow-up data were collected from the medical charts. Pre-hospital arrest data were collected with the use of a preformatted standard data collection tool.

The primary end-point of our study was to assess the valvular integrity of the left heart valves after Impella support. A secondary end point was used to observe any differences in the valvular integrity of the heart valves according to the initial setting of the patients’ admission (protected-PCI vs. shock) or device (Impella 2.5 vs. CP).

### 2.5. Statistical Analysis

All data were analyzed retrospectively. Data are presented as absolute variables and percentages (%) for categorical variables and either median with interquartile range (IQR: 25th–75th percentile) or mean with standard deviation according to the distribution of the variables. We assessed normality using Shapiro-Wilk, Pearson as well as Kolmogorov-Smirnov test. After testing for normal distribution, Student’s *t*-test or Mann-Whitney test was implemented to test for differences between the various characteristics. For categorical variables, Fisher’s exact test, chi-square, or the Friedman non-parametric test were used, as appropriate. All analyses were made using SPSS 24 and Graphpad Prism 6.0. A two-sided *p* < 0.05 was considered statistically significant.

## 3. Results

A total of 84 patients were enrolled in the study: 22 received an Impella in terms of a protected-PCI, where the remaining 62 were in profound CS. The etiology of CS comprised of 17 patients with acute myocarditis or decompensated dilative cardiomyopathy, 43 patients with acute coronary syndromes and subsequent coronary revascularization procedures and two patients with severe aortic stenosis (AS). Two patients underwent a balloon valvuloplasty before Impella implantation. Eight patients were resuscitated before admission, whereas no device was placed under ongoing cardiopulmonary resuscitation. The majority of patients (*n* = 60) received an Impella 2.5, while 24 of the patients received an Impella CP. The demographic characteristics of the participants are demonstrated on Table 1. The mean duration of Impella support was 5 days. As expected, the mean duration of Impella support was statistically significant longer in the CS group compared to protected-PCI group of patients, while there were not any differences observed between Impella 2.5 and Impella CP patients. The CS patients were younger compared to protected-PCI and they tended to be more obese, while the patients in the protected-PCI group had more previous kidney dysfunction. The average follow-up was 732 ± 310 days. Out of 295 patients with Impella in the index period, 165 patients died during the initial hospital stay. Follow-up echocardiographic data are available in only 83 of these patients, since the majority of patients expired directly or soon after ICU admission. There were only two cases of severe mitral insufficiency, mainly due to destruction of the mitral valve secondary to chordal rupture or valve leaflet perforation after Impella extraction. Both patients were admitted after out of hospital cardiac arrest and died due to severe septic shock with multi-organ failure. Out of the 130 patients discharged alive from the initial hospital stay, long-term follow up-data were available in 84 patients. Additional baseline and demographic data regarding the total of the patients of the index period are listed on Supplementary Table S1.

Overall, MR not only did not worsen on the course of time in any of the subgroups, but also it was observed a statistically significant reduction of the severe regurgitations. On baseline, there were eight patients with severe MR, while on follow-up there were not any cases with severe regurgitation (*p* = 0.007 between baseline and follow-up for outcome severe MR). The greatest benefit was demonstrated in the CS subgroup, while four cases of severe MR in the Impella 2.5 and four cases in the CP group were downgraded to moderate in follow–up. There were no cases of derangement of MV structural integrity, specifically no evidence of chordal rupture, papillary muscle rupture, valve perforation or prolapse across all time points. Similarly, there were not any changes observed in the MS on the course of time in all patients. A MV endocarditis was not observed in the patient cohort. Detailed data concerning the MV findings are listed on Table 2.

AV did not worsen across the time in any of the study groups. 2 patients on the protected-PCI group supported with Impella were observed to have marginally a moderate AR compared to mild on baseline, whereas two patients having a moderate AR in the CS group supported with Impella 2.5 seemed to have only a mild in follow-up. The incidence of AS was overall rather low, there were only two cases with a severe stenosis on baseline (both patients refused aortic valve replacement), while two cases with mild stenosis on baseline were upgraded to severe in the follow-up period. There were no cases of derangement of AV structural integrity, specifically no evidence of valve perforation or prolapse across all time points. There were not any cases of AV endocarditis. All data concerning the AV findings are demonstrated on Table 3.

**Figure 1:**
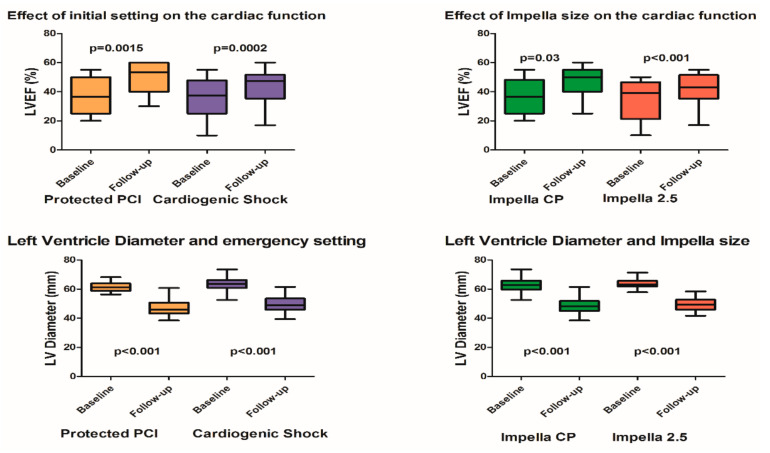
Impact of Impella support on the left ventricle function and diameter according to the initial setting and Impella size.

Our results showed that the cardiac function was in general terms significantly improved in the course of follow-up. Although there was not any statistically significant difference on the EF between baseline (37.3 ± 13.2%) and discharge (41.7 ± 12.3%) in the protected-PCI group, the improvement of the EF during follow-up (50.2 ± 10.53%) reached a great statistical significance (*p* = 0.0015) (Figure 1). In the CS group, the improvement of the EF reached a statistical significance upon discharge (41.25 ± 12.49% vs. 36.5 ± 12.59% on baseline, *p* = 0.03) and was even greater during follow-up (44.38 ± 10.52%, *p* = 0.0002) (Figure 1). Similarly, the EF was improved in the Impella 2.5 group already upon discharge (42.27 ± 11.68% vs. 37.5 ± 12.27%, *p* = 0.03) and the difference was even greater on follow-up (47.07 ± 10.53% vs. 37.5 ± 12.27%, *p* < 0.001), while in the CP group the EF demonstrated a statistically significant improvement only on follow-up (42.5 ± 10.84% vs. 34.67 ± 10.40%, *p* = 0.031). Additionally, it was shown significant architectural changes in LV leading to a significant decrease of the distension of the LV on the course of time in both settings (protected-PCI vs. shock and Impella 2.5 vs. CP) (Figure 1). There was no evidence of proximal aortic or LV structural damage.

Device related ischemic vascular complications occurred in 10 (11.9%) patients. These complications included six patients needing device extraction [4 patients with Impella 2.5 (6.7%) and two patients with Impella CP (8.3%), all patients with cardiogenic shock (9.7%)] and four patients requiring intervention ((percutaneous angioplasty (*n* = 2) or surgical repair (*n* = 2), all patients in the Impella 2.5 group (6.7%) and with cardiogenic shock (6.5%)). Access-site bleeding requiring transfusion occurred in 9 (10.7%) patients. None of our patients experienced an in-hospital or during follow-up stroke or myocardial reinfarction. None of the patients of the protected PCI group needed a hospitalization due to heart failure during follow-up, in five patients of the cardiogenic shock group a hospitalization was needed during follow-up, while two patients were admitted twice during follow-up. None of the patients underwent another ventricular assist device, while three (3.6%) patients underwent pacemaker implantation during initial stay and six (7.1%) patients needed a cardiac resynchronisation therapy during follow-up. Additional safety outcome data regarding the total patients of the index period are listed on Supplementary Table S1.

## 4. Discussion

This is a retrospective observational study evaluating the performance of Impella 2.5 and CP on left heart valvular integrity and LV function in two high risk patients’ groups—patients with CS and those who underwent protected-PCI. The main finding of our study is the absence of any negative structural changes on the left heart valves and secondarily the significant improvement of the LV function with a direct improvement of the LV architecture via a statistically significant reduction of the LV distension, a fact that in part is attributed to valvular integrity. In this outcome setting, we present for the first time a direct comparison of Impella 2.5 (12FR motor pump) with the larger Impella CP (14FR motor pump) showing similar result irrespectively of the device size in a so long follow-up period. The valvular structures of left heart remained intact periprocedural, at hospital discharge and at 4 months or longer after device implantation, as assessed with echocardiograms according to protocol.

In our study there were initially eight patients with severe MR, two in the protected-PCI group and 6 in the CS group (four in the Impella 2.5 group and four in the Impella CP group). Interestingly, all patients with severe MR at baseline showed significant improvement on the course of time with absence of any case of severe MR in the follow-up period. It is important to note that there have been isolated case reports of valve injury including MR secondary to chordal rupture or valve leaflet perforation [16,17], as well as a mild functional MS which resolved after removing the Impella [18]. In our center, the Impella is always positioned in the catheterization laboratory with the use of biplane angiography system paying special attention for the stabilization of LV inlet central avoiding lateral positions or contact with the MV apparatus. Moreover, the position of Impella is routinely controlled in the ICU with the use of echocardiography (transthoracic/transesophageal), especially before extraction of the device. However, the statistically significant improvement in the MR profile cannot be solely attributed to the mechanical unloading of the left ventricle, because the revascularization itself as well as the optimal medical treatment could clearly overlap such an effect. It is important to underline that the main goal of our study is to provide only an echocardiographic evaluation of structural integrity in a means of safety outcome and not to emphasize on the functional recovery of the left heart, which at the end may be multifactorial.

In line with the previous study from Goldstein et al., we did not detect any structural damage of the AV [13]. The direct crossing of the Impella through the AV has emerged concerns about the future function of the valve, especially about inflammatory mechanisms that could mediate a direct destruction of the valve architecture. AV is anatomically and pathophysiologically a passive ventile functioning only according to pressure differences. As such, the direct crossing of the valve may be associated with fewer risks as compared with other structures that dispose a stabilization apparatus. Among our study patients, there were only two cases with mild AR on baseline demonstrating an up-regulation in a moderate insufficiency in the follow-up study. This could be an incidental effect of the progression of the underlying disease mediating the AR; interestingly enough, there were not any cases of moderate of severe AR in the setting of the Impella CP, which deploys a thicker inlet in the left ventricle. Additionally, we observed two cases in the protected-PCI group receiving an Impella 2.5 with an up-regulation from mild to severe AS. These cases concern a 78- and an 82-year old patient with a follow-up period of 36 and 44 months respectively, meaning an advancement that cannot necessarily be attributed to the crossing of the valve with Impella, rather to a progression of valve calcification, as assessed from the echocardiograms. Interestingly, there were not any cases of AS progression in the setting of the thicker ventricular inlet Impella CP, implying that these cases observed in the previous Impella 2.5 group is rather a matter of incidence or the net effect of the underlying mechanisms of AV disease.

An interesting finding of our study is the statistically significant improvement of the EF and of the LV architecture in all subgroups between baseline and follow-up. This can and, of course, should be more attributed to direct benefit from the revascularization procedures as well as the positive remodeling through optimal medical treatment, eventual cardiac resynchronization therapy and absence of shock on discharge or in follow-up. In this study effort, we did not mean to point out or even to imply an improvement of the function of the left ventricle through the unloading of the Impella, since our aim was strictly refined to safety outcomes of this kind of mechanical support. In the same direction, the improvement of the ejection fraction as well as of the LV architecture could not be mediated, if the Impella had, as means of support, a devastating structural effect on the left heart valves of the LV. However, similar results were observed in previous studies, while Impella provides better unloading profile compared to optimal medical strategies in severe CS [5,13].

We report a 11.1% rate of vascular complications as well as an access-site bleeding requiring transfusion rate of 10.7% amomg our patients. Recently, a large-scale propensity-matched, registry-based retrospective cohort study observational including 1768 patients with CS treated with Impella reported high rates of major bleeding of 31.3%, which was markedly higher compared to the bleeding rates in our Impella cohort [19]. On the other side, further retrospective studies reported lower vascular complication rates comparable to our results [20,21]. Registry data including 112 patients supported with Impella for AMI-related CS reported an overall vascular complication rate of 17% with limb ischemia occurring in 3.5% and major access-site bleeding in 9.8% of patients [20]. Another retrospective analysis of 237 patients with AMI-associated CS treated with Impella reported peripheral ischemic vascular complications in 9.8% of Impella patients [21]. Under this prism, our safety outcome rates are concordant with previous real world Impella cohorts, depicting the clinical reality and everyday practice of our patient collective, although the relatively high device related rate of short-term complications as the natural selection due to short-term mortality has led to exclusion of patients of the assessed cohort.

Study Limitations

Our observations are obviously limited by the retrospective and non-randomized design of our study. However, this is the first and largest study so far to compare the effects of the Impella size on the valvular integrity of the left heart valves. Moreover, this is the first study with so long follow-up and the only effort till to date focusing on structural changes of left heart valves according to an elective or CS setting. Moreover, our investigation was a single-center study, and our study population was relatively selective with the majority of patients having myocardial infarction as cause of the CS. Therefore, the results should be interpreted in this context. However, coronary artery disease remains the leading cause of the CS. Additionally, the improvement of the EF or MR may well be associated with optimal medical treatment, cardiac resynchronization therapy or revascularization itself, so that a direct mediating effect of Impella unloading on the improvement of cardiac or functional status cannot be causally proven. Lastly, there were not any cases with Impella 5.0. However, this type of Impella needs surgical placement and assumes the existence of cardiothoracic support, meaning that it would be not always feasible in emergency settings or in centers lacking cardiothoracic departments. Given the retrospective nature of this study, the results remain preliminary and all above associations need to be evaluated in future accordingly designed studies.

## 5. Conclusions

In conclusion, Impella is a safe support method as far the structural integrity of the left heart valves in a long follow-up period is concerned. This finding was constant and irrespective of the initial indication for the mechanical support as well as of the size of the pump used. Whether Impella improves the cardiac function or not should be further evaluated in future studies.

## Figures and Tables

**Table 1:** Demographics and baseline characteristics of the study population.

	Total Group (*n* = 84)	Ptotected-PCI (*n* = 22)	Cardiogenic Shock (*n* = 62)	*p*-Value	Impella 2.5 (*n* = 60)	Impella CP (*n* = 24)	*p*-Value
Age, (years of age)	66.52 ± 12.24	72.36 ± 10.27	64.45 ± 12.28	0.01	67.5 [59, 75]	66.5 [60.8, 73.8]	0.4
Gender, male/female	69/15	18/4	51/11	1	49/11	20/4	1
BMI, kg/m^2^	26.81 ± 3.47	25.54 ± 3.39	27.26 ± 3.43	0.05	26.18 [24.22, 29.32]	26.4 [24.64, 27.69]	0.71
Etiology of cardiogenic shock	62 (73.8)	-		-	42 (70)	20 (83.3)	0.28
DCM/Myocarditis			17 (27.4)		7 (16.7)	10 (50)	
Myocardial Infarction			43 (69.4)		33 (78.6)	10 (50)	
Aortic Stenosis			2 (3.2)		2 (4.7)	0 (0)	
Duration of hospital stay (days)	18.29 ± 7.08	15.45 ± 4.75	19.29 ± 7.52	0.03	18 [17, 22.5]	16.93 ± 5.7	0.05
Duration of Impella support (days)	5 [2, 7]	2 [1, 6]	5 [3, 7]	0.001	4 [2, 7]	6 [5, 7]	0.05
**Medical comorbidities**							
Hypertension, *n* (%)	60 (71.4)	20 (91)	40 (64.5)	0.03	45 (75)	15 (62.5)	0.29
Diabetes, *n* (%)	24 (28.6)	6 (27.3)	18 (29)	1	19 (31.7)	5 (20.1)	0.43
PAD, *n* (%)	18 (21.4)	6 (27.3)	12 (20)	0.57	13 (21.7)	5 (20.1)	1
Prior stroke, *n* (%)	8 (9.5)	2 (9.1)	6 (9.7)	1	6 (10)	2 (8.3)	1
COPD, *n* (%)	18 (21.4)	4 (18.2)	14 (22.6)	0.77	7 (11.7)	11 (45.8)	0.002
Renal insufficiency (GFR < 60 mL/min), *n* (%)	18 (21.4)	10 (45.5)	8 (13)	0.005	12 (20)	6 (25)	0.77
Prior CAD, *n* (%)	26 (33.3)	6 (27.3)	20 (32.3)	0.79	17 (28.3)	9 (37.5)	0.44
Prior MI, *n* (%)	24 (28.6)	6 (27.3)	18 (29)	1	17 (28.3)	7 (29.2)	1
Prior PCI, *n* (%)	24 (28.6)	6 (27.3)	18 (29)	1	17 (28.3)	7 (29.2)	1
Prior CABG, *n* (%)	10 (11.9)	0 (0)	10 (16.1)	0.06	7 (11.7)	3 (12.5)	1

BMI: body mass index; ICU: intensive care unit; PAD: peripheral artery disease; COPD: chronic obstructive pulmonary disease; GFR: glomerular filtration rate; CAD: coronary artery disease; MI: myocardial infarction; PCI: percutaneous coronary intervention; CABG: coronary artery bypass graft; Numbers are presented as mean (± standard deviation), median (interquartile range: IQR 25th–75th percentile) or frequency (percentile).

**Table 2:** Mitral valve findings.

**(a) Mitral Valve Regurgitation Findings**								
	**Protected PCI (*n* = 22)**				**Cardiogenic Shock (*n* = 62)**			
	**Baseline**	**Discharge**	**Follow-Up**	***p***	**Baseline**	**Discharge**	**Follow-Up**	***p***
None or trivial/mild	18	18	20	0.14	46	46	54	<0.01
Moderate	2	2	2		10	8	8	
Severe	2	2	0		6	8	0	
	**Impella 2.5 (*n* = 60)**				**Impella CP (*n* = 24)**			
None or trivia/mild	48	50	54	<0.01	16	14	20	<0.01
Moderate	8	6	6		4	4	4	
Severe	4	4	0		4	6	0	
**(b) Mitral Valve Stenosis Findings**								
	**Protected PCI (*n* = 22)**				**Cardiogenic Shock (*n* = 62)**			
	**Baseline**	**Discharge**	**Follow-Up**	***p***	**Baseline**	**Discharge**	**Follow-Up**	***p***
None or trivial/mild	20	20	20	1	60	62	62	0.14
Moderate	2	2	2		2	0	0	
Severe	0	0	0		0	0	0	
	**Impella 2.5 (*n* = 60)**				**Impella CP (*n* = 24)**			
None or trivia/mild	58	58	58	1	0	0	0	1
Moderate	2	2	2		0	0	0	
Severe	0	0	0		0	0	0	

**Table 3:** Aortic valve findings.

**(a) Aortic Valve Regurgitation Findings**								
	**Protected PCI (*n* = 22)**				**Cardiogenic Shock (*n* = 62)**			
	**Baseline**	**Discharge**	**Follow-Up**	***p***	**Baseline**	**Discharge**	**Follow-Up**	***p***
None or trivial/mild	22	22	20	0.14	60	60	62	0.14
Moderate	0	0	2		2	2	0	
Severe	0	0	0		0	0	0	
	**Impella 2.5 (*n* = 60)**				**Impella CP (*n* = 24)**			
None or trivial/mild	58	58	58	1	24	24	24	1
Moderate	2	2	2		0	0	0	
Severe	0	0	0		0	0	0	
**(b) Aortic Valve Stenosis Findings**								
	**Protected PCI (*n* = 22)**				**Cardiogenic Shock (*n* = 62)**			
	**Baseline**	**Discharge**	**Follow-Up**	***p***	**Baseline**	**Discharge**	**Follow-Up**	***p***
None or trivial/mild	22	22	20	0.14	60	60	58	0.14
Moderate	0	0	2		0	0	0	
Severe	0	0	0		2	2	4	
	**Impella 2.5 (*n* = 60)**				**Impella CP (*n* = 24)**			
None or trivial/mild	56	56	54	0.14	24	24	24	1
Moderate	2	2	2		0	0	0	
Severe	2	2	4		0	0	0	

## Data Availability

The data presented in this study are available on request from the corresponding author. The data are not publicly available due to ethical restrictions.

## Supplementary Materials

The following are available online at https://www.mdpi.com/2077-0383/10/6/1273/s1, Table S1: Demographics, baseline characteristics and main outcomes of the study population.
