# Supplementary material for: Left Ventricle Architecture and Valvular Integrity Following Microaxial Mechanical Support: A Two-Year Follow-Up Study
Source: J Clin Med. 2021 Mar 18;10(6):1273. doi: 10.3390/jcm10061273 (PMC8003263; doi:10.3390/jcm10061273)
Supplement: Supplementary file 1 [file jcm-10-01273-s001.pdf]

Supplementary Table 1. Demographics, baseline characteristics and main outcomes of the study population

	**All patients (n=295)**	**Cardiogenic Shock (n=262)**	**Protected-PCI (n=33)**	**p**
Age, years	69.50 ± 11.91	69.02 ± 12.28	73.15 ± 7.82	0.06
Gender, male/female	230/65	205/57	25/8	0.82
BMI, kg/m^2	28.32 ± 5.76	28.40 ± 5.67	29.4 ± 5.4	0.34
Etiology of Impella support				
Acute myocardial Infarction, n (%)	*248 (79.1)*	*233 (88.9)*	*15 (45.4)*	*<0.01*
DCM/Myocarditis, n (%)	*29 (9.8)*	*29 (11.1)*	*0 (0)*	*0.06*
Protected-PCI, n (%)	*33 (11.1)*	*0 (0)*	*33 (100)*	----
Prior Cardiac Arrest, n (%)	105 (35.6)	105 (40)	0 (0)	<0.01
**Medical comorbidities**				
Hypertension, n (%)	196 (66.4)	170 (65)	26 (80)	0.12
Diabetes, n (%)	93 (31.5)	79 (30)	14 (42.4)	0.17

PAD, n (%)	55 (18.6)	47 (18)	8 (24.2)	0.35
Stroke, n (%)	28 (9.5)	25 (9.5)	3 (9.1)	1
Prior CAD, n (%)	142 (48.1)	118 (45)	24 (72.7)	<0.01
Prior MI, n (%)	84 (28.5)	63 (24)	21 (63.6)	<0.01
Prior PCI, n (%)	109 (36.9)	86 (32.8)	23 (69.7)	<0.01
Prior CABG, n (%)	32 (10.8)	23 (8.8)	9 (27.2)	<0.01
Charlson Comorbidity Index (age adjusted)	4.65 ± 2.31	4.62 ± 2.29	4.91 ± 2.42	0.07
**Hemodynamic variables**				
Dobutamine, n (%)<br>(μg/kg/min)	164 (62.6)<br>5.8 ± 2.39*	151 (57.6)<br>6.22 ± 2.34*	13 (39.3)<br>3.11 ± 1.6*	0.06<br><0.01
Norepinephrine, n (%)<br>(μg/kg/min)	262 (88.8)<br>0.27 ± 0.15	262 (100)<br>0.31 ± 0.12	0 (0)<br>----	----<br>----
Epinephrine, n (%)<br>(μg/kg/min)	60 (20.3)<br>0.08 ± 0.1	60 (22.9)<br>0.21 ± 0.05*	0 (0)<br>----	----<br>----

Heart rate (bpm)	93.54 ± 28.36	95.47 ± 29.01	78.21 ± 15.94	<0.01
Mean blood pressure (mmHg)	72.99 ± 18.14	71.84 ± 18.32	82.12 ± 13.68	<0.01
Lactate (mmol/L)	6.28 ± 4.07	6.68 ±3.96	1.27 ± 0.54**	<0.01
**Survival and safety outcomes**				
Survival to discharge, n (%)	130 (44.1)	107 (40.8)	23 (69.7)	<0.01
Etiology of death, n (%)				
Refractory shock or multi organ failure	*152 (51.5)*	*144 (55)*	*8 (24.2)*	*0.14*
Brain death	*8 (2.7)*	*8 (3.1)*	*0 (0)*	*0.6*
Impella-related complications	*5 (1.7)*	*3 (11.4)*	*2 (6)*	*0.1*
Access-site bleeding requiring transfusion, n (%)	28 (9.5)	26 (9.9)	3 (9.1)	1
Limb ischemia requiring intervention, n (%)	32 (10.8)	30 (11.4)	2 (6)	0.76
Myocardial Reinfarction, n (%)	3 (1)	3 (1.1)	0 (0)	1
Stroke, n (%)	6 (2)	6 (2.3)	0 (0)	1
Non-device-related bleeding, n (%)	3 (1)	3 (1.1)	0 (0)	1

PAD: peripheral artery disease; PCI: percutaneous coronary intervention; CABG: coronary artery bypass graft; DCM: dilative cardiomyopathy. Numbers are presented as mean (± standard deviation) or frequency (percentile).

* among patients with the index medication

** data available in only 23 of the patients
